# South West Food Community: A Place-Based Pilot Study to Understand the Food Security System

**DOI:** 10.3390/nu11040738

**Published:** 2019-03-29

**Authors:** Stephanie Louise Godrich, Jennifer Payet, Deborah Brealey, Melinda Edmunds, Melissa Stoneham, Amanda Devine

**Affiliations:** 1 School of Medical and Health Sciences, Edith Cowan University, Bunbury, WA 6230, Australia; j.payet@ecu.edu.au (J.P.); d.brealey@ecu.edu.au (D.B.); 2 Public Health Advocacy Institute of Western Australia, Curtin University, Bentley, WA 6102, Australia; melinda.edmunds@curtin.edu.au (M.E.); M.Stoneham@curtin.edu.au (M.S.); 3 School of Medical and Health Sciences, Edith Cowan University, Joondalup, WA 6027, Australia; a.devine@ecu.edu.au

**Keywords:** food security, public health, place-based, co-design

## Abstract

The objectives of this study were to: (i) Identify initiatives supporting healthy food availability, access and utilisation in the South West region of Western Australia (WA); and (ii) understand how they were functioning as a system to enhance community-level food security (FS). This study used a novel approach; a Systemic Innovation Lab, to interview initiative leaders/stakeholders about their FS initiative. Initiative characteristics measured included those which were associated with creating the effective conditions for FS systems change. Information was uploaded to an innovative online tool, creating a ‘transition card’ (matrix) of initiatives and partnering organisations. Fifty-one participants reported on 52 initiatives. Initiatives were most likely to possess characteristics relating to reinforcing changes towards an enhanced way of working to address FS and creating disruption to the old way of working. The initiative characteristic that initiatives were least likely to possess related to identifying the different causal factors of FS, and working with other stakeholders on specific components of FS. The South West Food Community pilot project used a comprehensive yet defined approach to demonstrate the value of a place-based, co-design project. Participants and stakeholders could strengthen specific initiative characteristics to facilitate enhanced community-level FS.

## 1. Introduction

Food security (FS) refers to sufficient physical, economic and social access to safe and nutritious food at all times [1]. At a community level, this extends to all community residents obtaining adequate food through a sustainable food system that maximizes self-reliance [2]. Suboptimal or inadequate food access results in food insecurity (FI) [3], which is known as a complex ‘wicked’ problem [4]. The unique issue is not well understood, nor resolved by any particular individual solution [5]. To facilitate understanding of the issue’s components, four pillars of FS are typically defined: Food availability, access, utilisation, and stability of the first three pillars [6,7,8]. Within these pillars, a range of determinants are described. Food availability drivers include well-located retail options selling affordable, nutritious food of sufficient quantity [9,10,11,12]. Determinants of food access include adequate resources to access food, private, public and active transport opportunities [9,10,12,13]. Within the food utilisation pillar, determinants include adequate food preparation, cooking and storage facilities, cultural considerations, nutrition knowledge and cooking skills to achieve physiological needs [9,10,12]. The stability of the first three pillars refers to the ability of all individuals, households and communities to have sustained access to nutritious food at all times [12], thus being food secure.

By and large, most Australians are thought to be food secure [10], with the reported household FI prevalence at 4% [14] nationally. However, other Australian research suggests this is an underestimate, with the prevalence reported to be as high as 36% [15]. FS can be particularly challenging to ensure, especially in rural and remote locations, given the poorer food availability, quality and higher cost [9,16,17]. Additional challenges associated with financial and physical access to food or nutrition knowledge and food preparation skills can hamper efforts to achieve FS [9,10,13,16]. Recommendations to enhance FS across food availability, access, utilisation and stability pillars have included local, sustainable food supply options, enhanced social support and increasing the number and duration of food literacy programs [9]. However, given the complexity of this issue, the need to move beyond viewing FS through pillars alone, and thus adopting a systemic approach, is necessary [18].

Approaches to increase FS systemically include the use of ‘lab’ approaches. These experimental methods identify societal issues, develop and test impactful ideas to solve such issues [19]. Labs use varying methods, focus areas, processes and typically possess one or more features identified in the literature to address wicked issues [19]. However, given the varied scope, their efficacy is limited. To more effectively address complex issues, a novel lab type coined a ‘Systemic Innovation Lab’ [20], can be used. This new, holistic lab type includes all elements recommended to address complex concepts, such as FS [20]. The process is based on a series of principles that create enabling conditions for adopting a more effective way of addressing complex problems [20]. Features include addressing the unpredictability of complex problems that have interconnected determinants [20,21], a place-based focus engaging a variety of stakeholders [18,19,22] transitioning to a new, more effective way of working [20,23], and self-organisation [20,24]. When these elements are collectively used, this approach facilitates place-based data collection and identifies governments’ enabling influences for change [20]. It also involves a range of stakeholders from various levels of government and community throughout the process [20]. Community participation or citizen engagement in health promotion projects ensures research meets community needs, increases public awareness, improves research translation and decision-making [25]. It also results in more effective solution generation, increases trust, empowers participants and increases the potential for proposed strategies or outcomes to be accepted by the wider community [26]. 

A critical component of the new Systemic Innovation Lab approach is the nine embedded Focus Areas, which align with the aforementioned principles to create enabling conditions for change. Specific Focus Areas include: (1) Create a disequilibrium state (shaking up the current way of working); (2) Amplify action (transitioning towards a new and better way of working); (3) Encourage self-organisation (organisations working in new ways); (4) Stabilise feedback (locking in the new way of working); (5) Enable information flows (disseminating information throughout the system); (6) Unplanned exploration at the interface between a government bureaucracy and the community (aligning community organisations’ work with government priorities); (7) Unplanned exploration at the interface between the elected members of a government and the community (community organisations shaping government policies); (8) Planned exploitation at the interface between the community and a government bureaucracy (government using community knowledge and ideas); and (9) Planned exploitation at the interface between the community and the elected members of a government (government sharing information about community initiatives). Further detail about these Focus Areas and their characteristics has been previously published [18,27].

Whilst previous research has investigated the determinants and the prevalence of household FI in Western Australia (WA) [9,13,16,17,28], a systemic examination of FS at a community level has yet to be conducted. We currently lack a broad understanding of the initiatives being used to improve FS in WA. We also lack a comprehensive map of these initiatives across regions, local government areas and communities. Further, existing partnerships that support these initiatives are yet to be identified. This highlights a clear gap in evidence and practice, and hampers potential action to enhance FS in WA. The resultant piece-meal approach impedes the understanding and improvement of FS, particularly in rural and remote areas where food system challenges are heightened. The objectives of this pilot project were therefore to: (i) Identify the initiatives supporting healthy food availability, access and utilisation in South West WA; and (ii) understand how they are functioning as a system to enhance FS.

## 2. Materials and Methods 

### 2.1. Sampling and Recruitment

This pilot study took place in the South West region of WA, south of Perth and covers 23,970 km^2^ [29] and includes inner regional and outer regional towns [30]. The project utilised a Systemic Innovation Lab [18] approach, which was based on appropriate features for addressing complex issues such as FS [20]. This process, developed by the organisation Wicked Lab (www.wickedlab.com.au), aligned with systemic design and was underpinned by complex systems leadership theories and complex adaptive systems [18,27]. The methodology used, including the steps of Form, Explore, Map, Learn, Address and Share (FEMLAS) [20], is explained in detail elsewhere [20] and thus a summary is presented in this paper. A cross-institutional core team and reference group provided guidance, feedback and oversight of project processes. Structured individual or group interviews were deemed the most appropriate data collection method, for FS initiatives and their characteristics. This approach facilitated assessment of alignment between initiatives’ characteristics and those associated with transitioning to a more effective way of increasing FS [18]. 

A Microsoft Excel database was compiled by the project team via an Internet (Google) search of programs and projects (initiatives) operating in the selected geographical boundary; the South West region of WA. To be included in the database, initiatives had to focus on one or more FS pillars in the South West region of WA. Within these pillars, initiatives were required to address one or more of the determinants of FS, for example healthy food promotion, social support, nutrition knowledge and cooking skills [6,10]. Example search terms included a broad scope such as ‘food security programs in South West Western Australia’. Search terms also related to FS pillars, such as ‘food availability in South West Western Australia’, and FS determinants such as ‘social support programs South West Western Australia’ and ‘nutrition education programs South West Western Australia’. The initiative database included the initiative name, a description, start and finish date (if ceased), initiative owner/stakeholder name, email address, website URL and partnering organisations involved with the initiative. Interview participants were identified as project coordinators, staff, volunteers or committee members working on one or more initiatives. In addition, database contacts were added from existing stakeholder network databases. An organisation database included the administering organisation’s name, a description of the organisation, sector (i.e., business, not-for-profit organisation) and website URL. 

To increase stakeholder buy-in, recruitment and to identify additional initiatives, stakeholders from state and local government, community organisations and community members were invited to a project launch. During this launch, the proposed process was outlined and participants were engaged in a scoping activity to outline the current South West region initiatives focusing on the FS pillars. The purpose of this activity was to familiarise attendees with the local FS system and identify further potential initiatives for inclusion. Identified stakeholders from the Internet search and project launch (*n* = 79) were invited by email to participate in an interviewer-administered survey and sent an information letter and consent form. A minimum of three follow up contacts were made to recruit participants. Of these, 51 stakeholders consented to participate in an interview (65% response rate).

### 2.2. Instrument

A 45-item survey tool was developed by the project team and was linked to Wicked Lab’s Tool for Systemic Change [31], a digital tool designed specifically to address wicked problems. The Tool for Systemic Change was based on a model focusing on 36 initiative characteristics embedded within the nine aforementioned Focus Areas. These Focus Areas and their characteristics were associated with creating desirable conditions for systems change; transitioning to a more effective way of enhancing FS [18,20]. Each Focus Area characteristic was linked to a survey question. The survey was cross-checked by Wicked Lab consultants to ensure the questions retained the intent of the Focus Area characteristics. An example of a Focus Area 1 characteristic included ‘cultivate a passion for action.’ [18]. The related interview question was “Does your initiative create a passion for the community to take action around food security? (i.e. encouraging/influencing the system to a new way of working, such as community getting involved with creating a local edible garden)”. See Supplementary Table S1 for a complete list of Focus Areas, their associated characteristics and survey interview questions. Response options to questions included ‘Yes’ or ‘No’. Participants were asked to provide a comment on why they believed that their initiative did or did not contain this characteristic. Additional survey items included demographic questions such as worker type, years working in the field, a description of their initiative/s and partner organisations with whom they were working.

### 2.3. Data Collection

#### Interviews

Qualitative interviews were the chosen data collection method, and are deemed appropriate for the investigation of novel concepts or issues [32]. Interviews afford a greater understanding of such issues [33]. Data collection occurred between July–October 2018 by two interviewers. Individual or group interviews using the survey tool were conducted in person (*n* = 3) or via telephone (*n* = 38) with a total of 51 participants. To ensure rigour and consistency, both interviewers co-conducted the first interview, which commenced with a verbal preamble about the study purpose, format and number of questions. Interviewees were advised there were no correct or incorrect answers; the responses were based on their perception of their initiative. Interviewers proceeded to ask all questions to respondents, taking notes during each interview and digitally entering responses into a Microsoft Excel spreadsheet thereafter.

### 2.4. Data Entry and Analysis

**Figure 1:**
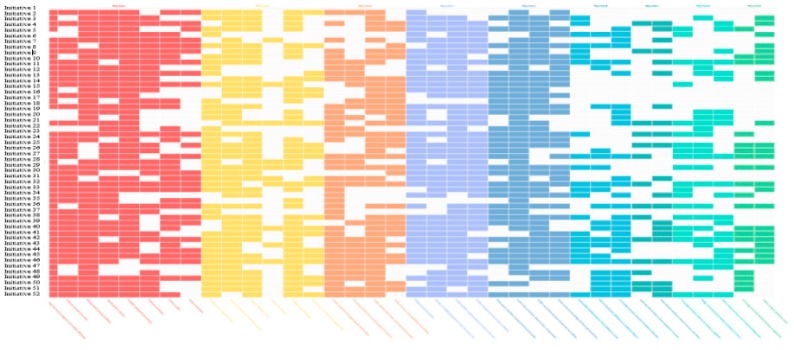
South West WA Food Community ‘transition card’ at baseline mapping.

Microsoft Excel was used to manage interview data before being uploaded to Wicked Lab’s Tool for Systemic Change [18,31]. The Tool for Systemic Change captured information about the organisation that owned the initiative, contact details, any partnering organisations working on the initiative and details about the length of time the initiative had been operating. The Tool for Systemic Change also included a series of tick boxes relating to each Focus Area and their characteristics, which were ticked where the initiative met the characteristics [20]. In addition, text boxes captured open-ended responses explaining how the initiative did or did not possess the characteristic. This enabled the creation of a visual ‘transition card’ (matrix) displaying the initiatives and the characteristics they possessed, within the nine Focus Areas (Figure 1) [18]. If the initiative possessed the characteristic within the Focus Area, the cell on the transition card corresponding to the tick box response was filled. This provided a visual representation of each initiative’s contribution to systemic change [20]. In addition, it displayed how the ‘solution ecosystem’ of initiatives and their partner organisations collectively contributed towards systemic change in the South West WA region. The transition card was subsequently analysed initiative by initiative and as a whole, to highlight gaps in opportunity that could be harnessed to improve FS across the region. Reports generated in Microsoft Excel format by the Wicked Lab tool included descriptive statistics of: The total number of initiatives; total number of partnering organisations; number of organisations by sector (i.e., business, not for profit); the number of partner organisations per initiative; and number of initiative per organisation [18]. A report also provided the number and proportion (%) of initiatives that met each Focus Area’s characteristics. Initiatives were then summed by FS pillar in an additional report; categorised into ‘food availability’, ‘food access’ or ‘food utilisation’ pillars. Food availability initiatives were categorised where they aligned with the definition of “sufficient quantities of food of appropriate quality, supplied through domestic production, imports” [34]. This included food sourced through formal or informal means, such as through community gardens. It incorporated initiatives that aligned with one or more of the food availability pillar determinants of food availability, food price, location of outlets, food quality, promotion, or food variety [6,9,10]. An example includes a health and wellbeing plan supporting local agricultural development, with the purpose of increasing healthy food availability. Food access was defined as “the resources and ability that communities, households and individuals have in order to acquire and consume a healthy diet” [34]. Initiatives were categorised within this pillar if they aligned with one or more of its determinants of social support, household finances, transport, distance to outlets and mobility [6,9,10]. An example initiative included a fresh produce swapping group open to any community member. The food utilisation definition included “utilisation of food through adequate diet, clean water, sanitation to reach a state of nutritional wellbeing, where all physiological needs are met [34].” Initiatives were categorised within this pillar if they aligned with one or more of its determinants of nutrition knowledge and cooking skills, food preferences, storage or cooking facilities or time to purchase and prepare food [6,9,10]. An example initiative is a community strategy that supports nutrition education.

After results were reviewed by the project team, participants were invited together in a results-sharing and action planning forum. Forum participants (*n* = 20) were provided with a second briefing paper which outlined the process taken and a copy of the overall transition card. In addition, each participant was provided with an individual summary report for their initiative/s, to facilitate understanding of strengths and ‘windows of opportunity’ to strengthen. A facilitated action-planning session with provision of examples allowed participants to develop initiative action plans, which outlined strategies to fill identified gaps and enhance their initiative/s functioning to address FS. Participants also discussed potential new FS initiatives, which could be explored to fill identified gaps in the system. A workshop video recording and Microsoft Power Point slides were sent along with individual action plans to participants not able to attend the workshop. 

All participants gave their informed consent for inclusion before they participated in the study. The study was conducted in accordance with the Declaration of Helsinki of 1975, revised in 2013, and the protocol was approved by the Edith Cowan University Human Research Ethics Committee (project 20508).

## 3. Results

### 3.1. Participant Demographics

A total of 41 individual or group interviews were conducted, with 51 participants. Interviewees were most often volunteers, volunteer leaders or committee members (*n* = 13), followed by directors, managers or coordinators (*n* = 11). Participants had worked, on average, three years in their field. Table 1 presents a demographic profile of study participants’ worker type. 

### 3.2. Partnering Organisations

There were 83 partnering organisations working on identified initiatives. The majority of interviewees reported partnering with not-for-profit groups (37%), businesses (24%) and state government organisations (19%) to deliver their initiative/s. Table 1 outlines the frequency of partnering organisation type.

### 3.3. Initiative Characteristics

A total of 52 initiatives were captured in the mapping process. Initiatives were categorised by FS pillar, with 32 initiatives relating to the ‘food availability’ pillar. Example initiatives within this pillar included farmers’ markets, food trails, local government plans and agritourism initiatives. Thirty initiatives related to the ‘food access’ pillar and included produce swapping groups, community gardens and community plans outlining objectives or strategies to increase community food access. A total of 31 initiatives were categorised within the ‘food utilisation’ pillar and were provided by way of community nutrition education initiatives, educational farm stays and other examples including fundraising activities through FS awareness-raising events. No initiatives were categorised in the ‘stability’ pillar. Most initiatives focused on a combination of food availability, access and/or utilisation dimensions. 

### 3.4. Focus Areas and Initiative Characteristics

Overall, initiatives were most likely to possess characteristics within Focus Area 4, ‘stabilise feedback’, which related to reinforcing progression to an enhanced way of working (Table 2). This was followed by Focus Area 1, ‘create a disequilibrium state’, which related to disrupting the old/previous way of working. The initiative characteristic met by the majority of initiatives (92%) included the Focus Area 1 characteristic ‘highlight the need to organise communities differently’. This characteristic related to the initiative encouraging communities to address FS in a new or innovative way. Equally, 92% of initiatives reportedly possessed the Focus Area 5 characteristic ‘assist in the connection, dissemination and processing of information’. This was achieved either through a newsletter, website or through social media. 

The initiative characteristic that was least likely to be met included the Focus Area 2 characteristic of ‘partition the system’ (*n*= 10, 19%). This could include identifying the different causal factors (parts) of FS and working with other stakeholders on specific parts of FS. An example provided to participants that exemplified this characteristic included a working group made up of members of various sectors, collectively working towards a strategy. This was followed by only 14% of initiatives addressing the characteristic of ‘assist elected members to frame policies in a manner which enables community adaptation of policies’. An example provided to participants included a local government putting a call out for community ideas supporting healthy food access through social media.

Table 2 depicts the frequency and proportion of initiatives that met Focus Area characteristics. Results were determined by interviewee responses to the survey questions that linked to each characteristic. A detailed explanation of these characteristics has been published elsewhere [27]. 

Figure 1 provides a visual representation of the ‘transition card’ of initiatives (y-axis) and their associated Focus Area characteristics (x-axis). The visual representation identifies gaps and opportunities from a set of unlinked community-based initiatives. As seen by the uncoloured cells, and as presented in Table 2, initiatives were least likely to possess characteristics within Focus Areas 6–9, which focused on the community–government interface. Example characteristics included government staff working with community groups through a committee, or investigating how the government could support initiatives that were addressing the objectives in the government Strategic Plan. Each initiative owner received an individual summary report highlighting their associated row within Figure 1. 

## 4. Discussion

The objectives of this project were to: (i) Identify the initiatives supporting healthy food availability, access and utilisation in South West WA; and (ii) understand how they were functioning as a system to enhance FS. This pilot project resulted in the identification of 52 initiatives in the South West solution ecosystem. Of these, 32 initiatives related to healthy food availability, 30 focused on food access and 31 initiatives focused on food utilisation. Most initiatives focused on a combination of availability, access and/or utilisation dimensions. To deliver their initiatives, organisations partnered with state and local government, as well as the not-for-profit and business sectors. Initiatives were investigated to determine presence of characteristics associated with addressing complex problems, and encouragingly, many communities addressed FS in a new or different way. Information dissemination about FS was also a common attribute, and the majority of initiatives possessed this characteristic. Initiatives were least likely to partition the FS system, for example working with cross-sector stakeholders to address components of FS, or achieve the Focus Areas of 8 and 9—the interface with public administration and elected members. 

Previous literature has demonstrated the value of encouraging communities to take a new approach to address complex problems [23,27], to disrupt the system; a characteristic that the majority of initiatives in this project possessed. Similarly, the importance of ensuring information flows throughout the system, whilst it is transitioning from old to new approaches has been reinforced [23,27]. Most of the initiatives captured in this project shared information through the FS system via newsletters or electronic means. Previous evidence has reported the value of stakeholder engagement opportunities afforded by social networking sites, such as awareness raising, sharing program success, or support fundraising efforts [35]. Although less than half of the initiatives possessed the characteristic ‘partition the system’, previous evidence has acknowledged this characteristic is imperative to support stakeholders to be co-creators of initiatives [20,36]. The value of co-creation lies in its facilitation of needs-based research design, effective decision-making and solution generation, community empowerment and resultant action [25,26].

This project has answered the call of previous research, contributing to a greater understanding of how FI can be addressed as a wicked problem through the use of a Systemic Innovation Lab approach [18]. The approach utilised in this project has supported the South West FS system of organisations and initiatives to identify where their initiatives are functioning effectively to address FS. It also assisted participants to identify where changes were required within their initiatives, to incorporate characteristics within specific areas to leverage change. For example, an outcome of participating in the action-planning forum could include a Local Government Authority hosting a community consultation to review their proposed Public Health Plan, to ensure it meets their needs associated with healthy food availability, access and use. This change would result in the initiative possessing characteristics associated with creating policies that the community shape. Another example could include a community group providing their local government staff with a solution template about FS, explaining how their initiative supports it. This action would result in the community initiative possessing a characteristic associated with sharing information about community initiatives. If multiple initiatives changed their practice and incorporated strategies that linked to associated characteristics they were previously lacking, the system of initiatives would have transitioned to a new and more effective way of supporting FS. Thus, resulting in systemic change and enhancing community-level FS in the region [20]. In addition, the project has used steering strategies to create opportunities for government and community stakeholders to interact, resulting in the collaborative design of adaptations to existing and potential new FS initiatives. For example, cross-pollination of ideas and advice between such stakeholders at the action-planning forum. Therefore, supporting enhanced FS governance [18]. However, further research is now required to evaluate changes to initiatives that occur as a result of the Systemic Innovation Lab approach [20]. This work will involve interviewing initiative leaders to ascertain changes to initiative characteristics and then recreating the transition card to measure impact on the food security system. In addition, conducting further research on a larger scale would likely ensure initiatives addressing the ‘stability’ FS pillar are captured and would facilitate region-to-region comparisons.

This project possessed a number of strengths, such as a more rigorous evaluation of initiatives’ capacity to transition towards enhancing FS. A known limitation of FS initiatives to date has been limited assessment of this impact [37]. Another strength was the innovative Systemic Innovation Lab [20] approach used to support multiple initiatives to transition to a more effective way of enhancing FS [20]. By taking a solution ecosystem approach, the complexity associated with FS was considered [20]. The place-based nature of the project also enabled the identification of synergies within the local context and across social, economic, environmental and cultural dimensions [20]. The use of a common platform to connect the various stakeholders participating in the project was another strength [20,38], which increased stakeholder awareness of the characteristics their initiative/s possessed, as well as the opportunities for improvement. The engagement of these stakeholders as co-creators [20] was another strength. However, the pilot project is not without limitations. This project included a small sample of stakeholders (*n* = 51), limiting its generalisability and also limiting the capture of all existing initiatives in the South West region of WA. Secondly, given the variability in respondents’ roles, varying levels of details about initiatives were captured. In addition, this project did not involve verification of respondents’ comments about initiative characteristics with document analysis or collection of any other program information. Thirdly, the transitioning to a more effective way of working may not have been the intention of the organisations, and the possession of the desired characteristics may have been by chance. This project required translation from complex academic language associated with theories that underpinned the constructs, to plain English. Two team members had participated in a six-month Complex Systems Leadership Program [39], delivered by Wicked Lab and focusing on the constructs that underpinned the project model. Therefore, these team members led the development of the survey tool used in interviews as well as crosschecking the tool with Wicked Lab’s consultants, to ensure language used was appropriate for all participants whilst retaining the integrity of the model. However, the other project team members had not participated in the training. Full team member training would likely facilitate enhanced understanding of project concepts among participating stakeholders. Finally, as this project was a pilot, a limited number of initiatives were uncovered in the discrete project timeframe. This resulted in no identification of initiatives addressing the ‘stability’ pillar of FS. Though it remains unknown whether such initiatives exist in the region.

## 5. Conclusions

FS focuses on ensuring physical, social and economic access to food. While FS in rural and remote locations can be challenging to sustain, the South West region provided numerous opportunities to increase community-level FS. This pilot project investigated FS strategies being implemented across the South West region of WA. It mapped their initiative characteristics to desirable characteristics using an online tool. This comprehensive but defined approach demonstrated the value of a place-based co-design approach to addressing FS.

## Figures and Tables

**Table 1:** Participant Demographics.

**Worker type**	***n* (%)**
Education professional	1 (2)
Food producer/farmer	6 (11)
Environmental health officer	1 (2)
Community development officer, services worker or support officer	7 (13)
Director, manager or coordinator	11 (21)
Volunteer, volunteer leader or committee member	13 (24)
Health professional (i.e., health promotion officer, nutritionist, dietitian, nurse)	9 (17)
CEO or President	2 (4)
Social enterprise manager	1 (2)
Sustainability or recycling officer	2 (4)
**Total**	53 * (100)
**Partner organisation**	***n* (%)**
Local government	9 (11)
State Government	16 (19)
Education	1 (1)
Not-for-profit	31 (37)
Business	20 (24)
Formal community group	3 (4)
Informal community group	3 (4)
**Total**	**83 (100)**

* Some participants performed more than one role in their initiative/s.

**Table 2:** Frequency of initiatives meeting Focus Areas and characteristics.

Focus Areas and Characteristics Met by Initiatives	*n* (%)
Focus Area 1: Create a disequilibrium state (Shaking up the current way of working)	
Highlight the need to organise communities differently	48 (92)
Cultivate a passion for action	39 (75)
Manage initial starting conditions	46 (88)
Specify goals in advance	43 (83)
Establish appropriate boundaries	46 (88)
Embrace uncertainty	34 (65)
Surface conflict	34 (65)
Create controversy	25 (48)
Focus Area 2: Amplify action (moving to a new and better way of working)	
Enable safe fail experimentation	41 (79)
Enable rich interactions in relational spaces	42 (81)
Support collective action	38 (73)
Partition the system	10 (19)
Establish network linkages	40 (77)
Frame issues to match diverse perspectives	21 (40)
Focus Area 3: Encourage self-organisation (organisations working in new and more effective ways with each other)	
Create correlation through language and symbols	40 (77)
Encourage individuals to accept positions as role models for the change effort	27 (52)
Enable periodic information exchanges between partitioned subsystems	40 (77)
Enable resources and capabilities to recombine	31 (60)
Focus Area 4: Stabilise feedback (the new way of working becomes the dominant way of working among the organisations in the system).	
Integrate local constraints	42 (81)
Provide a multiple perspective context and system structure	39 (75)
Enable problem representations to anchor in the community	47 (90)
Enable emergent outcomes to be monitored	42 (81)
Focus Area 5: Enable information flows (helping to get information spread throughout the system)	
Assist system members to keep informed and knowledgeable of forces influencing their community system	34 (65)
Assist in the connection, dissemination and processing of information	48 (92)
Enable connectivity between people who have different perspectives on community issues	40 (77)
Retain and reuse knowledge and ideas generated through interactions	34 (65)
Focus Area 6: Public administration–adaptive community interface (Helping the work undertaken by community organisations to align with government priorities)	
Assist public administrators to frame policies in a manner which enables community adaptation of policies	21 (40)
Remove information differences to enable the ideas and views of citizens to align to the challenges being addressed by governments	22 (42)
Encourage and assist street level workers to take into account the ideas and views of citizens	28 (54)
Focus Area 7: Elected government–adaptive community interface (Creating government policies that are shaped by community organisations)	
Assist elected members to frame policies in a manner which enables community adaptation of policies	14 (27)
Assist elected members to take into account the ideas and views of citizens	21 (40)
Focus Area 8: Community innovation–public administration interface (Government using community knowledge and ideas)	
Encourage and assist street level workers to exploit the knowledge, ideas and innovations of citizens	20 (38)
Bridge community-led activities and projects to the strategic plans of governments	23 (44)
Gather, retain and reuse community knowledge and ideas in other contexts	21 (40)
Focus Area 9: Community innovation–elected government interface (The government sharing information about community initiatives operating in their area)	
Encourage and assist elected members to exploit the knowledge, ideas and innovations of citizens	20 (38)
Collect, analyse, synthesise, reconfigure, manage and represent community information that is relevant to the electorate or area of portfolio responsibility of elected members	24 (46)

## Supplementary Materials

The following are available online at https://www.mdpi.com/2072-6643/11/4/738/s1, Table S1: Focus Areas, embedded characteristics, and associated survey interview questions.

## References

[1] Food and Agriculture Organization Rome Declaration on World Food Security and World Food Summit Plan of Action. http://www.fao.org/docrep/003/w3613e/w3613e00.HTM.

[2] Hamm M.W., Bellows A.C. (2003). Community food security: background and future directions. J. Nutr. Educ. Behav..

[3] Household Food Insecurity in Canada, 2011. Research to Identify Policy Options to Reduce Food Insecurity (PROOF). https://proof.utoronto.ca/resources/proof-annual-reports/annual-report/.

[4] Grochowska R. (2014). Specificity of food security concept as a wicked problem. JAST.

[5] Conklin J. (2006). Dialogue Mapping: Building Shared Understanding of Wicked Problems.

[6] Food Security: The What, How, Why and Where to of Food Security in NSW: Discussion Paper. https://ses.library.usyd.edu.au/handle/2123/9082.

[7] An Introduction to the Basic Concepts of Food Security. www.fao.org/docrep/013/al936e/al936e00.pdf.

[8] Bach C., Aborisade B. (2014). Assessing the Pillars of Sustainable Food Security. Eur. Int. J. Sci.Technol..

[9] Godrich S.L., Davies C.R., Darby J., Devine A. (2017). What are the determinants of food security among regional and remote Western Australian children?. Aust. N. Z. J. Public Health.

[10] Food Security Options Paper: A Planning Framework and Menu of Options for Policy and Practice Interventions. https://www.health.nsw.gov.au/heal/Publications/food-security.pdf.

[11] Lawlis T., Islam W., Upton P. (2018). Achieving the four dimensions of food security for resettled refugees in Australia: A. systematic review. Nutr. Diet..

[12] Food Security. http://www.fao.org/fileadmin/templates/faoitaly/documents/pdf/pdf_Food_Security_Cocept_Note.pdf.

[13] Pollard C., Nyaradi A., Lester M., Sauer K. (2014). Understanding food security issues in remote Western Australian Indigenous communities. Health Promot. J. Aust..

[14] 4364.0.55.009-Australian Health Survey: Nutrition-State and Territory results, 2011-12. http://www.abs.gov.au/ausstats/abs@.nsf/Lookup/4364.0.55.009main+features12011-12.

[15] Butcher L.M., O’Sullivan T.A., Ryan M.M., Lo J., Devine A. (2018). Utilising a multi-item questionnaire to assess household food security in Australia. Health Prom. J. Aust..

[16] Pollard C., Landrigan T., Ellies P., Kerr D., Lester M., Goodchild S. (2014). Geographic Factors as Determinants of Food Security: A Western Australian Food Pricing and Quality Study. Asia Pac. J. Clin. Nutr..

[17] Pollard C., Savage V., Landrigan T., Hanbury A., Kerr D. (2015). Food Access and Cost Survey.

[18] Zivkovic S. (2017). Addressing food insecurity: A systemic innovation approach. Soc. Enterprise J..

[19] Innovation Teams and Labs, a Practice Guide. https://media.nesta.org.uk/documents/innovation_teams_and_labs_a_practice_guide.pdf.

[20] Zivkovic S. (2018). Systemic Innovation Labs: A lab for wicked problems. Soc. Enterprise J..

[21] Snowden D.J., Boone M.E. (2007). A leader’s framework for decision making. Harv. Bus. Rev..

[22] The Evaluation of Place-Based Approaches. https://www.horizons.gc.ca.

[23] Goldstein J., Hazy J., Lichtenstein B. (2010). Complexity and the Nexus of Leadership.

[24] Sorensen E., Torfing J. (2017). Metagoverning collaborative innovation in governance networks. Am. Rev. Public Adm..

[25] Consumers Health Forum of Australia Statement on Consumer and Community Involvement in Health and Medical Research. https://www.nhmrc.gov.au/sites/default/files/documents/reports/consumer-community-involvement.pdf.

[26] Developing Effective Citizen Engagement: A How-to Guide for Community Leaders. http://www.rural.palegislature.us/effective_citizen_engagement.pdf.

[27] Zivkovic S. (2015). A complexity based diagnostic tool for tackling wicked problems. Emerg. Complex. Organ..

[28] Godrich S.L., Lo J., Davies C.R., Darby J., Devine A. (2017). Prevalence and socio-demographic predictors of food insecurity among regional and remote Western Australian children. Aust. N. Z. J. Public Health..

[29] Our WA Regions. http://www.drd.wa.gov.au/regions/Pages/default.aspx.

[30] Australian Statistical Geography Standard (ASGS). http://www.abs.gov.au/websitedbs/d3310114.nsf/home/australian+statistical+geography+standard+(asgs).

[31] A Tool for Systemic Change. http://www.wickedlab.com.au/tool-for-systemic-change.html.

[32] Corbin J., Strauss A. (2009). Basics of Qualitative Research: Techniques and Procedures for Developing Grounded Theory.

[33] Jamshed S. (2014). Qualitative research method-interviewing and observation. J.Basic Clin. Pharm..

[34] Agriculture and Development Economics Division. http://www.fao.org/economic/agricultural-development-economics/en/.

[35] Waters R.D., Burnett E., Lamm A., Lucas J. (2009). Engaging stakeholders through social networking: How nonprofit organizations are using Facebook. Public Relat. Review..

[36] Surie G., Hazy J.K. (2006). Generative leadership: Nurturing innovation in complex systems. Emerg. Complex. Organ..

[37] Collins P.A., Power E.M., Little M.H. (2014). Municipal-level responses to household food insecurity in Canada: A call for critical, evaluative research. Can. J. Public Health.

[38] State of the Future. http://www.millennium-project.org/publications-2-3/.

[39] Zivkovic S. Determining and increasing the performance of a Complex Systems Leadership Program. Proceedings of the 9th International Social Innovation Research Conference.

