# Supplementary material for: South West Food Community: A Place-Based Pilot Study to Understand the Food Security System
Source: Nutrients. 2019 Mar 29;11(4):738. doi: 10.3390/nu11040738 (PMC6521197; doi:10.3390/nu11040738)
Supplement: Supplementary file 1 [file nutrients-11-00738-s001.pdf]

Table S1: Focus Areas, embedded characteristics, and associated survey interview questions

Focus Areas and characteristics met by initiatives	**Example interview question**
**Focus Area 1: Create a disequilibrium state (Shaking up the current way of working)**	
Highlight the need to organise communities differently	Does your initiative encourage communities to address food security in a new or innovative way? (i.e. using a newsletter to highlight a vision of the government in the future)
Cultivate a passion for action	Does your initiative create a passion for the community to take action around food security? (i.e. encouraging/influencing the system to a new way of working, such as community getting involved with creating a local garden)
Manage initial starting conditions	Have you managed the initial starting conditions of your initiative, such as through consultation to shape the project?
Specify goals in advance	Does your initiative have a clear project outline or broad goals that were identified from project commencement and allow scope for adjustment?
Establish appropriate boundaries	Does your initiative operate within defined boundaries? It might be geographical, for example a neighbourhood, local government region or electorate. Or it could be a boundary based on the challenge it aims to address, for example goals in a community or strategic plan.
Embrace uncertainty	Does your initiative highlight that there is no predetermined plan or outcome that it is trying to achieve? This could include program participants reflecting on their own beliefs about an issue and develop innovative ideas to solve the issue.
Surface conflict	Does your initiative expose conflict? Examples include working in a different way to how the community operates, or differences in understanding between community members. This could be enhanced by increasing community member awareness that there are a number of conflicting perspectives on how to address a particular issue, such as food insecurity, which could be considered.
Create controversy	Does your initiative create controversy? For example, does your initiative highlight alarming facts about the problem? Does your initiative highlight the need for public debate, discussion or contention about the problem? This could involve media to create publicity about an issue.
**Focus Area 2: Amplify action (moving to a new and better way of working)**	
Enable safe fail experimentation	Does your initiative allow for 'safe to fail' experimentation? For example, approaching a problem/issue in a small, contained way that allows unplanned outcomes to be seen. The ones that produce desired outcomes are adopted and those which don't are allowed to fail.
Enable rich interactions in relational spaces	Does your initiative include the bringing together of a variety of stakeholders and opening up discussion, encouraging differences that foster new ideas to emerge and to utilise collective knowledge?
Support collective action	Does your initiative support collective action? The more that collective action is supported in the community, the more likely that actions will be amplified quickly throughout the community system and meaningful change will be achieved. I.e. bringing together stakeholders from different backgrounds to come up with a common agenda to solve food security.
Partition the system	Does your initiative support the breaking up of the work into different focus areas? This can include identifying the different casual factors (parts) of food security and working with other stakeholders on specific parts of food security? For example, a working group made up of various sectors collectively working towards a strategy.
Establish network linkages	Does your initiative establish networks? These networks need to be made with partners/collaborators that have the necessary expertise, contacts, and resources to support expanded activities. I.e. setting up a database of stakeholders, food security ecosystem map or project register.
Frame issues to match diverse perspectives	Does your initiative frame food security issues that match diverse perspectives? This encourages the connection with people, groups and organisations that have different points of view on the issue. I.e. writing discussion guides on an issue and bringing together participants at forums to work through issues.

**Focus Area 3: Encourage self-organisation (organisations working in new and more effective ways with each other)**	
Create correlation through language and symbols	Does your initiative create a connection through language and symbols? (i.e. tagline, branding). This can create a shared understanding of the system, where system members use the same language, and group communication is experienced by individual system members as if it is personalised.
Encourage individuals to accept positions as role models for the change effort	Does your initiative encourage individuals to accept positions as role models for the change effort? This supports the coherence required for the food security system and community to form a new pattern of working. I.e. relatable community members being invited to join a Community Taskforce.
Enable periodic information exchanges between partitioned subsystems	Does your initiative enable information exchanges between various stakeholders? For example, four different working groups working towards a collective volunteering strategy.
Enable resources and capabilities to recombine	Does your initiative enable resources and capabilities to recombine? For example, teams forming and sharing resources (i.e. computers or a building) and capabilities (i.e. staff skills) in relation to a project working on food security.
**Focus Area 4: Stabilise feedback (the new way of working becomes the dominant way of working among the organisations in the system).**	
Integrate local constraints	Does your initiative work around local constraints? This might mean adapting the initiative to be in line with local needs or the context in which it operates. Undertaking a Strengths, Weaknesses, Opportunities and Threats (SWOT) analysis can assist with identifying local constraints to projects.
Provide a multiple perspective context and system structure	Does your initiative allow people to identify food security problems in new ways, from different perspectives? Partnerships between community, business and government increases creativity and stops initiatives from working in isolation from one another.
Enable problem representations to anchor in the community	Does your initiative provide a variety of resources to increase awareness of what it does, and enable the community to participate and give their feedback? I.e. by providing research results back to the community, or community consultation for food security projects.
Enable emergent outcomes to be monitored	Is your initiative monitored for emerging outcomes? For example, watching to see how people's expectations of an initiative match up, or how different people explain an initiative's aims, goals and how it operates.
**Focus Area 5: Enable information flows (helping to get information spread throughout the system)**	
Assist system members to keep informed and knowledgeable of forces influencing their community system	Does your initiative allow stakeholders to keep informed and knowledgeable of things that influence food security? I.e. holding a seminar, or developing a toolkit to increase people's understanding of factors that impact an issue. This helps them address the issue.
Assist in the connection, dissemination and processing of information	Does your initiative assist in the sharing of information (i.e. through a newsletter, website or via social media)?
Enable connectivity between people who have different perspectives on community issues	Does your initiative enable people who have different perspectives on community issues to connect? I.e. Your organisation may have a volunteering strategy that is made up of representatives from local government, volunteers, community organisations.
Retain and reuse knowledge and ideas generated through interactions	Have you collated information about your initiative that is accessible to the community? I.e. a website that has a project register (list) of community projects addressing food insecurity.

**Focus Area 6: Public administration – adaptive community interface (Helping the work undertaken by community organisations to align with government priorities)**	
Assist public administrators to frame policies in a manner which enables community adaptation of policies	Does your initiative assist public administrators to frame policies in a manner which enables community adaptation of policies? (I.e. a Local Government Authority putting a call out for community ideas supporting healthy food access, through Facebook. These ideas would then be included in the Local Government Authority's Public Health Plan).
Remove information differences to enable the ideas and views of citizens to align to the challenges being addressed by governments	Does your initiative enable the ideas and views of community members to align with the challenges being addressed by governments? (i.e. by a Local Government Authority including food security information or their strategic plan on their website. This helps initiatives addressing food insecurity become aware of the Local Government Authority's policy and can increase the potential for the initiative and local government views on the issue to be shared).
Encourage and assist street level workers to take into account the ideas and views of citizens	Does your initiative encourage and assist government staff who have direct contact with community members to take into account their ideas? (i.e. government staff working together with community workers on a committee, or through a workshop)
**Focus Area 7: Elected government – adaptive community interface (Creating government policies that are shaped by community organisations)**	
Assist elected members to frame policies in a manner which enables community adaptation of policies	Does your initiative assist elected members to write or talk about policies in a way that allows the community to change them? (I.e. a community project that started because of the way a policy was talked about in the media).
Assist elected members to take into account the ideas and views of citizens	Does your initiative assist elected members to consider the ideas of community members? (I.e. community development training of local elected members in a Local Government Authority, to help them to take into account community member ideas).
**Focus Area 8: Community innovation – public administration interface (Government using community knowledge and ideas)**	
Encourage and assist street level workers to exploit the knowledge, ideas and innovations of citizens	Does your initiative encourage and assist government workers who work directly with community members to use the knowledge and ideas of community members? (I.e. an initiative that has a job description which includes Key Performance Indicators around including community feedback OR an initiative that uses a Theory of Change Template to demonstrate their initiative's goals/inputs).
Bridge community-led activities and projects to the strategic plans of governments	Does your initiative bridge community-led activities and projects to the strategic plans of governments? (I.e. investigating how a Local Government Authority could support initiatives that are addressing the objectives in their strategic plan, OR including a government target as one of the initiative's targets).
Gather, retain and reuse community knowledge and ideas in other contexts	Does your initiative gather, retain and reuse community knowledge and ideas in other contexts? (I.e. having a food security project/initiative register on a government website).
**Focus Area 9: Community innovation – elected government interface (The government sharing information about community initiatives operating in their area)**	
Encourage and assist elected members to exploit the knowledge, ideas and innovations of citizens	Does your initiative encourage and assist elected members to use the knowledge, ideas and innovations of community members? (I.e. by giving the briefing paper on food security (prepared through South West Food Community) to elected members in your Local Government Authority).
Collect, analyse, synthesise, reconfigure, manage and represent community information that is relevant to the electorate or area of portfolio responsibility of elected members	Does your initiative collect and utilise community information that is relevant to the Local Government area? (I.e. a project register on the government website that is relevant to the Local Government area).
